# Entropy Inequalities for Lattices

**DOI:** 10.3390/e20100784

**Published:** 2018-10-12

**Authors:** Peter Harremoës

**Affiliations:** Copenhagen Business College, Nørre Voldgade 34, 1358 Copenhagen K, Denmark; harremoes@ieee.org; Tel.: +45-39-56-41-71

**Keywords:** conditional independence, entropy function, functional dependence, lattice, non-Shannon inequality, polymatroid function, subgroup, 94A17, 06B99

## Abstract

We study entropy inequalities for variables that are related by functional dependencies. Although the powerset on four variables is the smallest Boolean lattice with non-Shannon inequalities, there exist lattices with many more variables where the Shannon inequalities are sufficient. We search for conditions that exclude the existence of non-Shannon inequalities. The existence of non-Shannon inequalities is related to the question of whether a lattice is isomorphic to a lattice of subgroups of a group. In order to formulate and prove the results, one has to bridge lattice theory, group theory, the theory of functional dependences and the theory of conditional independence. It is demonstrated that the Shannon inequalities are sufficient for planar modular lattices. The proof applies a gluing technique that uses that if the Shannon inequalities are sufficient for the pieces, then they are also sufficient for the whole lattice. It is conjectured that the Shannon inequalities are sufficient if and only if the lattice does not contain a special lattice as a sub-semilattice.

## 1. Introduction

The existence of non-Shannon inequalities has received much attention since the first inequality of this type was discovered by Zhang and Yeung [1]. The basic observation is that any four random variables X, Y, *Z* and *W* satisfy the following inequality:

(1) $$2I\left(Z;W\right)\leq I\left(X;Y\right)+I\left(X;Z⊎W\right)+3I\left(Z;W|X\right)+I\left(Z;W|Y\right).$$

Here, C⊎D denotes the random variable that takes a value of the form $\left(c,d\right)$ if c=C and d=D. As usual, $I\left(\cdot ;\cdot \right)$ and $I\left(\cdot ;\cdot |\cdot \right)$ denote mutual information and conditional mutual information given by:

(2) $$\begin{array}{cc} I\left(X;Y\right)= & H\left(X\right)+H\left(Y\right)-H\left(X⊎Y\right), \end{array}$$

(3) $$\begin{array}{cc} I\left(X;Y|Z\right)= & H\left(X⊎Z\right)+H\left(Y⊎Z\right)-H\left(X⊎Y⊎Z\right)-H\left(Z\right). \end{array}$$

where *H* denotes the Shannon entropy. The inequality (1) is non-Shannon in the sense that it cannot be deduced from the positivity, monotonicity and submodularity of the entropy function on the variables X,Y,Z, and their joins, i.e., satisfaction of the following inequalities:

(4) $$\begin{array}{ccc} Positivity & H\left(X\right) & \geq 0\;, \end{array}$$

(5) $$\begin{array}{ccc} Monotonicity & H\left(X⊎Y\right) & \geq H\left(X\right), \end{array}$$

(6) $$\begin{array}{ccc} Submodularity & H\left(X⊎Z\right)+H\left(Y⊎Z\right) & \geq H\left(X⊎Y⊎Z\right)+H\left(Z\right)\;. \end{array}$$

Positivity and monotonicity were recognized by Shannon [2], while submodularity was first observed by McGill [3]. It is easy to show that any inequality involving only three variables rather than four can be deduced from Shannon’s inequalities [4]. The powerset of four variables is a Boolean algebra with 16 elements, and any smaller Boolean algebra corresponds to a smaller number of variables, so in a trivial sense, the Boolean algebra with 16 elements is the smallest Boolean algebra with non-Shannon inequalities.

In the literature on non-Shannon inequalities, all inequalities are expressed in terms of sets of variables and their joins. Another way to formulate this is that the inequalities are stated for the free ∪-semi-lattice generated by a finite number of variables. In this paper, we will also consider intersections of sets of variables. We note that for sets of variables, we have the inequality:

(7) $$I\left(X;Y|Z\right)\geq H\left(X\cap Y|Z\right).$$

Inequality (7) has even inspired some authors to use $I\left(\cdot \wedge \cdot \right)$ as notation for mutual information.

Although non-Shannon inequalities have been known for two decades, they have found remarkably few applications compared with the Shannon inequalities. One of the reasons is that there exists much larger lattices than the Boolean algebra with 16 elements for which the Shannon inequalities are sufficient. The simplest examples are the Markov chains:

(8) $$X_{1}\to X_{2}\to X_{3}\to \cdots \to X_{n}$$

where any variable $X_{j}$ is determined by its predecessor, i.e., the conditional entropies $H\left(X_{j+1}|X_{j}\right)$ are zero for j=1,2,⋯,n−1. For such a chain, one has:

(9) $$H\left(X_{1}\right)\geq H\left(X_{2}\right)\geq H\left(X_{3}\right)\geq \cdots \geq H\left(X_{n}\right)\geq 0.$$

The inequalities (9) are all instances of the entropy function being monotone, and it is quite clear that these inequalities are sufficient in the sense that for any sequence of values that satisfies these inequalities, there exists random variables related by a deterministic Markov chain with these values as entropies.

In this paper, we look at entropy inequalities for random variables that are related by functional dependencies. Functional dependencies give a partial ordering of sets of variables into a lattice. Such functional dependence lattices have many applications in information theory, but in this paper, we will focus on determining whether a lattice of functionally-related variables can have non-Shannon inequalities. In order to achieve interesting results, we have to restrict our attention to special classes of lattices.

Entropy inequalities have been studied using matroid theory, but finite matroids are given by geometric lattices, i.e., atomistic semi-modular lattices (see the textbook of Stern [5] for definitions). For the study of non-Shannon inequalities, it is more natural to look at general lattices rather than geometric lattices because many important applications involve lattices that are not atomistic or not semi-modular. For instance, a deterministic Markov chain gives a lattice that is not atomistic. It is known that a function is entropic if and only if it is (approximately) equal to the logarithm of the index of a subgroup in a group [6]. Therefore, it is natural to study entropic functions on lattices and their relations to subgroup lattices.

In this paper, we bridge lattice theory, database theory and the theory of conditional independence, but sometimes, the terminology in these fields does not match. In such cases, we give preference to lattice theory over database theory and preference to database theory over the theory of conditional independence. For instance, there is a property for closure operators that is called extensivity in the theory of lattices. We translate extensivity into a property for functional dependence, and it turns out that extensivity can be used instead of the property for functional dependences, which is called augmentation. Extensivity is apparently a weaker condition than augmentation, but together with the properties called monotonicity and transitivity, they are equivalent on finite lattices. Finally, we translate extensivity from functional dependencies to separoid relations that model the concept of conditional independence. In the literature on conditional independence, extensivity has been termed “normality” without any explanation why this term is used. We called it extensivity because it is equivalent to the notion of extensivity in lattice theory, which we consider as a more fundamental theory.

The paper is organized as follows. In Section 2, we describe the link between lattice theory and the theory of functional dependences in detail. We demonstrate how properties of closure operators associated with sub-semilattices correspond to the properties of functional dependence that are normally called Armstrong’s axioms. In Section 3, we describe positive monotone submodular functions (polymatroid functions) and how they lead to separoid relations on lattices. These separoid relations generalize the notion of conditional independence known from Bayesian networks and similar graphical models. We demonstrate how properties of separoid relations correspond to properties of functional dependences.

In Section 4, we describe entropy functions on lattices and how they correspond to subgroup lattices of a group. We conjecture that the Shannon inequalities are sufficient for describing entropic polymatroid functions of a lattice if and only if the lattice does not contain a special lattice as a sub-semilattice. In Section 5, we develop some technical results related to “gluing” lattices together. The gluing technique is very useful for planar lattices, and in Section 6, we demonstrate that entropic functions on planar modular lattices can be described by Shannon’s inequalities.

We finish with a short discussion, where we outline some future research directions. There is one appendix with some additional comments related to Armstrong’s axioms. These are mainly intended for readers that are familiar with the theory of functional dependencies in databases. A second appendix contains a long list of lattices that are used to document that polymatroid functions on lattices with seven or fewer elements can be described by Shannon’s inequalities.

Some of the results presented in this paper have been published in preliminary form and without proof [7,8], but since then, most of the results have now been strengthened or reformulated. In this paper, all proof details will be given.

## 2. Lattices of Functional Dependence

In this section, we shall briefly describe functional dependencies and their relation to lattice theory. The relation between functional dependence and lattices has been studied [7,9,10,11,12,13]. The relation between lattices and functional dependencies is closely related to minimal sets of Shannon-type inequalities [14,15]. Relations between functional dependencies and Bayesian networks have also been described [8,16]. Many problems in information theory and cryptography can be formulated in terms of functional dependencies.

**Figure 1:**
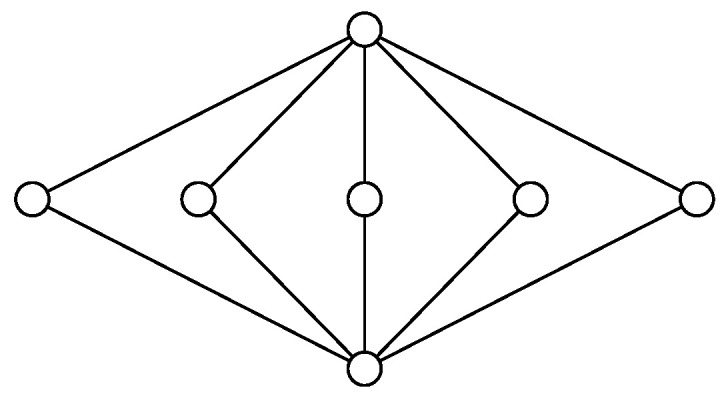
Hasse diagram of the lattice $M_{n}$ for n=5.

**Example** **1.** *Consider a group consisting of n agents. One might be interested in giving each agent in the group part of a password in such a way that no single agent can recover the whole password, but any two agents are able to recover the password. Here, the password should be a function of the variables known by any two agents, but must not be a function of a variable held by any single agent. The functional dependence structure is the lattice illustrated in the Hasse diagram in Figure 1. The node at the top illustrates the password. Each of the intermediate nodes represents the knowledge of an agent. The bottom node represents no knowledge.*

A ∧-semilattice is a set equipped with a binary operator ∧ that satisfies the following properties:

(10) $$\begin{array}{ccc} Commutativity & X\wedge Y & =Y\wedge X\;, \end{array}$$

(11) $$\begin{array}{ccc} Associativity & \left(X\wedge Y\right)\wedge Z & =X\wedge \left(Y\wedge Z\right)\;, \end{array}$$

(12) $$\begin{array}{ccc} Idempotency & X\wedge X & =X\;. \end{array}$$

For a ∧-semilattice the relation X∧Y=X defines a preordering that we will denote X≤Y. If $\left(L,\wedge \right)$ is a semilattice, then we say that M is sub-semilattice if M is closed under the ∧ operation. Let $\left(L,\wedge \right)$ denote a semilattice. Let ↓X={Y∈L∣Y≤X}. Then, $↓\left(X\wedge Y\right)=\left(↓X\right)\cap \left(↓Y\right)$. Therefore, we can identify any finite semilattice with a ∩-semilattice in a powerset. Since we will usually identify semilattice elements with sets of variables, we will often use ⊆ and ∩ to denote the ordering and the meet operation.

In this paper, we will assume that all semilattices and all lattices are finite. If a ∩-semilattice $\left(L,\cap \right)$ has a maximal element, then a binary operator ∨ can be defined as:

(13) $$X\vee Y=\underset{\begin{array}{c} Z⊇X\\ Z⊇Y \end{array}}{⋂}Z$$

and then, $\left(L,\cap ,\vee \right)$ is a lattice.

Let $\left(L,\subseteq \right)$ denote a lattice with M as a sub-semilattice with the same maximal element as L. Then, a unary operator cl:L→L can be defined by:

(14) $$cl\left(X\right)=\underset{\begin{array}{c} Z⊇X\\ Z\in M \end{array}}{⋂}Z$$

The operator cl is a closure operator [17], i.e., it satisfies:

(15) $$\begin{array}{cc} Extensivity & X\subseteq cl\left(X\right)\;, \end{array}$$

(16) $$\begin{array}{cc} Monotonicity & X\subseteq Y\;\mathrm{implies}\;cl\left(X\right)\subseteq cl\left(Y\right)\;, \end{array}$$

(17) $$\begin{array}{cc} Idempotency & cl\left(cl\left(X\right)\right)=cl\left(X\right)\;. \end{array}$$

For any closure operator cl, the element *X* is said to be closed if $cl\left(X\right)=X$. If *X* and *Y* are closed, then X∩Y is closed ([18], [Lemma 28]), so the closed elements of a lattice under a closure operator form a ∩-semilattice.

**Proposition** **1.** *Let $\left(L,\subseteq \right)$ denote a finite lattice. Assume that a subset M of L is closed under the meet operation and has the same maximal element as L. Then, M is a lattice under the ordering ⊆ with the meet operation in M given by ∩ and join operation in M given by $X⊎Y=cl\left(X\cup Y\right)$.*

**Example** **2.** *If G is a group, then a subgroup is defined as a subset that is closed under the group operations. The closure of a subset of G is the subgroup generated by the subset. The lattice of subgroups forms a ∩-semilattice in the lattice of all subsets of the group. Let G denote a finite group. For any subgroup $\tilde{G}\subseteq G$, we associate the variable $X_{\tilde{G}}$ that maps an element g∈G into the left coset $g\tilde{G}.$ Then, the subgroup lattice of G is mapped into a lattice of variables where the subset ordering of subgroups is equivalent to functional dependences between the corresponding variables.*

**Proposition** **2.** *If cl is a closure operator on a lattice, then the relation cl(X)⊇Y and the relation cl(X)⊇cl(Y) are equivalent. The relation X→Y given by cl(X)⊇Y satisfies the following properties.*

(18) $$\begin{array}{cc} Extensivity & X\to Y\;\mathrm{implies}\;X\to X⊎Y\;, \end{array}$$

(19) $$\begin{array}{cc} Monotonicity & X⊇Y\;\mathrm{implies}\;X\to Y\;, \end{array}$$

(20) $$\begin{array}{cc} Transitivity & \mathrm{If}\;X\to Y\;\mathrm{and}\;Y\to Z,\;\mathrm{then}\;X\to Z\;. \end{array}$$

**Remark** **1.** *The monotonicity of → is called reflexivity in the literature on databases. We reserve the notion of reflexivity to the relation X→X, in accordance with the terminology for ordered sets. In database theory, the property X→X is called self determination.*

*In the literature on databases extensivity, (18) is replaced by an apparently stronger property called augmentation, but in a finite lattice augmentation can be proven from extensivity, monotonicity and transitivity. See Appendix A for details.*

If the properties (18)–(20) are satisfied, we say that the relation → satisfies Armstrong’s axioms [19]

**Proof.**  Assume that $cl\left(X\right)⊇cl\left(Y\right)$. Using extensivity (15), we get cl(Y)⊇Y. The transitivity of ⊇ then gives cl(X)⊇Y.

Assume cl(X)⊇Y. Then, the monotonicity (16) gives cl(cl(X))⊇cl(Y), and the idempotent gives cl(X)⊇cl(Y).

To prove the extensivity (18) of →, assume that cl(X)⊇Y. Using the extensivity (15), we also get cl(X)⊇X. Combining these two inequalities gives cl(X)⊇X⊎Y, as desired.

The monotonicity (19) of → follows directly from the monotonicity (15) of cl.

The transitivity (20) of → follows from the transitivity of ⊇. □

If L is a lattice with a relation → that satisfies Armstrong’s axioms, then we say that a lattice element *X* is → closed if X→Y implies that X⊇Y.

**Theorem** **1.** *Let L be finite lattice with a relation → that satisfies Armstrong’s axioms. Then, the set of → closed elements form a ∩-semilattice with the same maximal element as L. The relation X→Y holds if and only if $cl\left(X\right)⊇Y$, where cl denotes the closure operator with respect to the semilattice.*

**Proof.**  Assume that $X_{1}$ and $X_{2}$ are closed and that $X_{1}\cap X_{2}\to Y.$ The monotonicity (19) implies $X_{i}\to X_{1}\cap X_{2}$, and then, the transitivity (20) implies that $X_{i}\to Y.$ Since $X_{i}$ is closed, we have $X_{i}⊇Y.$ Since this holds for both i=1 and i=2, we have $X_{1}\cap X_{2}⊇Y$, implying that $X_{1}\cap X_{2}$ is closed. The monotonicity (19) also implies that the maximal element of L is closed so that the set of closed elements M forms a ∩-semilattice with a closure operator $cl_{M}$.

Let cl denote the closure with respect to M. We will prove that $X\to cl\left(X\right)$. Let $X_{1}=X$. Assume that $X_{1}$ is not → closed. Then, there exists $Y_{1}$ such that $X_{1}\to Y_{1}$ and $X_{1}⊉Y_{1}$. Using the extensivity (18), we get $X_{1}\to X_{1}⊎Y_{2}$. Define $X_{2}=X_{1}⊎Y_{1}$. Then, $X_{1}\to X_{2}$ and $X_{1}\subset X_{2}$. Iterate this construction so that:

(21) $$\begin{array}{cc} X_{1} & \to X_{2}\to \cdots \to X_{n}\;, \end{array}$$

(22) $$\begin{array}{cc} X_{1} & \;\subset \;X_{2}\;\subset \;\cdots \subset \;X_{n}\;. \end{array}$$

Since the lattice is finite, the construction must terminate, and when it terminates, $X_{n}$ is closed. Using transitivity, we get $X\to X_{n}$ and $X\subseteq X_{n}$. Since $cl\left(X\right)$ is the smallest closed element greater than *X*, we have $X\to cl\left(X\right)$.

If $cl\left(X\right)⊇Y$, then $cl\left(X\right)\to Y$ by monotonicity (19), and then, X→Y by transitivity (20). If X→Y, then $cl\left(X\right)\to Y$. Using that $cl\left(X\right)$ is → closed, we get $cl\left(X\right)⊇Y$. □

We will look at functional dependencies in databases. Assume that a set of records is labeled by elements in a set *A*. In statistics records are the individual elements of a sample. For each record a∈A, the database contains the values of various attributes given by a number of functions from *A* to the set of possible attributes. Sets of such functions will be denoted by capital letters, and these will be our variables. We say that *X* determines *Y* and write X→Y if there exists some function *f* such that Y(a)=f(X(a)) for any record a∈A. Then, the relation → satisfies Armstrong’s axioms. Armstrong proved that these axioms form a complete set of inference rules [19]. That means that if a set *A* of functional dependencies is given and a certain functional dependence X→Y holds in any database where all the functional dependencies in *A* hold, then X→Y holds in that database. Therefore, for any functional dependence X→Y that cannot be deduced using Armstrong’s axioms, there exists a database where the functional dependence is violated [20,21]. As a consequence, there exists a database where a functional dependence holds if and only if it can be deduced from Armstrong’s axioms. Using the result that Armstrong’s axioms are equivalent to the closed sets forming a lattice, Armstrong’s result is easy to prove.

**Theorem** **2.** *For any finite lattice L, there exists a database with a set of related variables such that the elements of the lattice corresponds to closed sets under functional dependence.*

**Proof.**  As the set of records, we take the elements of the lattice L. With each Y∈L, we associate a function $f_{Y}:L\to L$ given by $f_{Y}\left(X\right)=Y\cap X$. If $Y_{1}⊇Y_{2}$, then:

(23) $$\begin{array}{cc} f_{Y_{2}}\left(X\right) & =Y_{2}\cap X\\ & =Y_{2}\cap \left(Y_{1}\cap X\right)\\ & =f_{Y_{2}}\left(f_{Y_{1}}\left(X\right)\right) \end{array}$$

so that $f_{Y_{2}}=f_{Y_{2}}\circ f_{Y_{1}}$. Therefore, $f_{Y_{1}}\to f_{Y_{2}}$.

Assume that $f_{Y_{1}}\to f_{Y_{2}}$. Let $X_{1}=Y_{1}$ and $X_{2}=Y_{1}⊎Y_{2}$. Then, $f_{Y_{1}}\left(X_{1}\right)=f_{Y_{1}}\left(X_{2}\right)=Y_{1}$, while $f_{Y_{2}}\left(X_{1}\right)=Y_{1}\cap Y_{2}$ and $f_{Y_{2}}\left(X_{2}\right)=Y_{2}$. Using that $f_{Y_{1}}\to f_{Y_{2}}$, we get $Y_{1}\cap Y_{2}=Y_{2}$, so that $Y_{1}⊇Y_{2}$. □

We have seen that for a subgroup lattice of a group, there exists a lattice of functional dependence. The opposite is also true. To each database with attributes related by functional dependence, there is a group. The construction is as follows. Let *A* denote a set of records. Let G=Sym(A) be the symmetric group consisting of permutations of the records. If *X* is a function on *A*, then we define the stabilizer group $G_{X}$ as the set of permutations that leave *X* invariant, i.e., permutations π∈Sym(A) such that X(π(a))=X(a) for all a∈A. Then, X→Y if and only if $G_{X}\subseteq G_{Y}$. In this way, the functional dependence lattice of a database can be mapped into a lattice of subgroups of a group.

Combining Theorem 2 with the stabilizers subgroups of the symmetric group of a database, we get the following result that was first proven in 1946 by Whitman [22].

**Corollary** **1.** *Any finite lattice can be represented as a functional dependence lattice generated by subgroups of a group.*

## 3. Polymatroid Functions and Separoids

**Definition** **1.** *On a lattice, the submodularity of a function h is defined via the inequality $h\left(X\right)+h\left(Y\right)\geq h\left(X⊎Y\right)+h\left(X\cap Y\right)$. If the submodular inequality holds with equality, we say that the function is modular. A polymatroid function on a lattice is a function that is non-negative, increasing and sub-modular.*

**Example** **3.** *Let L be finite atomistic lattice with a ranking function r:L→R. Then, L is a geometric lattice if and only if the function r is polymatroid ([5], Corollary 1.9.10).*

For a polymatroid function *h* on a lattice, one may introduce a function $I_{h}\left(\cdot ;\cdot |\cdot \right)$ that corresponds to conditional mutual information by:

(24) $$I_{h}\left(X;Y|Z\right)=h\left(X⊎Z\right)+h\left(Y⊎Z\right)-h\left(X⊎Y⊎Z\right)-h\left(Z\right).$$

One can rewrite $I_{h}\left(\cdot ;\cdot |\cdot \right)$ as:

(25) $$\begin{array}{c} I_{h}\left(X;Y|Z\right)=h\left(X⊎Z\right)+h\left(Y⊎Z\right)-h\left(X⊎Y⊎Z\right)-h\left(\left(X⊎Z\right)\cap \left(Y⊎Z\right)\right)\\ \;+h\left(\left(X⊎Z\right)\cap \left(Y⊎Z\right)\right)-h\left(Z\right). \end{array}$$

Since *h* is monotone and submodular, we have:

(26) $$\begin{array}{ccc} Positivity & I_{h}\left(X;Y|Z\right) & \geq 0. \end{array}$$

It is straightforward to verify that:

(27) $$\begin{array}{ccc} Symmetry & I_{h}\left(X;Y|Z\right) & =I_{h}\left(Y;X|Z\right)\;, \end{array}$$

(28) $$\begin{array}{ccc} Chain\;rule & I_{h}\left(X;Y⊎Z|W\right) & =I_{h}\left(X;Y|W\right)+I_{h}\left(X;Z|Y⊎W\right)\;. \end{array}$$

We will say that a function I(·;·∣·) that satisfies positivity (26), symmetry (27) and the chain rule (28) is a separoid function.

**Proposition** **3.** *If $I\left(\cdot ,\cdot |\cdot \right)$ is a separoid function, then the following property is satisfied.*

(29) $$\begin{array}{ccc} Monotonicity & Y\subseteq Z\;\mathrm{implies}\;I\left(X;Y|Z\right) & =0\;. \end{array}$$

**Proof.**  Assume that Y⊆Z. We can use the chain rule (28) to get:

(30) $$\begin{array}{cc} I\left(X;Y|Z\right) & =I\left(X;Y⊎Y|Z\right)\\ & =I\left(X;Y|Z\right)+I\left(X;Y|Y⊎Z\right)\\ & =2\cdot I\left(X;Y|Z\right)\;. \end{array}$$

Hence, monotonicity (29) is satisfied. □

The relation $I_{h}\left(X;X|Z\right)=0$ is equivalent to $h\left(X⊎Z\right)=h\left(Z\right)$, and this relation will be denoted $X\to_{h}Z$. The first to observe that $to_{h}$ defines a lattices was Shannon, who published a very short paper on this topic in 1953 [23]. Shannon did not mention the relation to the theory of functional dependences because that theory was only developed two decades later. Surprisingly, Shannon’s paper was only cited once until 2002!

The relation $\to_{h}$ satisfies Armstrong’s axioms, and the most instructive way to see this is via separoid relations. If *h* is a polymatroid function, then the relation $I_{h}\left(X,Y|Z\right)=0$ will be denoted $X{⊥\;⊥}_{h}Y|Z$. Following Dawid et al. [24,25], we say that a relation $\left(\cdot ⊥\;⊥\cdot |\cdot \right)$ on a lattice $\left(L,\cap ,⊎\right)$ is a separoid relation, if it has the following properties:

(31) $$\begin{array}{cc} Monotonicity & Y\subseteq Z\;\mathrm{implies}\;X⊥\;⊥Y|Z\;, \end{array}$$

(32) $$\begin{array}{cc} Symmetry & X⊥\;⊥Y|Z\;\mathrm{implies}\;Y⊥\;⊥X|Z\;, \end{array}$$

(33) $$\begin{array}{cc} Chain\;rule & X⊥\;⊥Y⊎Z|W,\;\mathrm{if}\;\mathrm{and}\;\mathrm{only}\;\mathrm{if}\;X⊥\;⊥Y|W\;\mathrm{and}\;X⊥\;⊥Z|Y⊎W\;. \end{array}$$

**Remark** **2.** *The term monotonicity was used for a different concept by Paolini [26]. In [24,25], a weaker condition than monotonicity was used, but their condition together with the chain rule implies monotonicity.*

With this definition we see that ${⊥\;⊥}_{h}$ is a separoid relation. The properties (31)–(33) should hold for all X,Y,Z,W∈L. In this paper, we are particularly interested in the case where the subsets are not disjoint. In the literature on Bayesian networks and similar graphical models, the focus has been on disjoint sets where only the last two properties (32) and (33) are used to define a semi-graphoid relation [27]. See also [28], Remark 2.5, where it is noted that semi-graphoid relations can be defined on join semi-lattices.

A long list of properties for the notion of independence was given by Paolini [26], but Studený has proven that one cannot deduce all properties of statistical conditional independence from a finite list of axioms [28,29].

**Proposition** **4.** *A separoid relation $\left(\cdot ⊥\;⊥\cdot |\cdot \right)$ on a lattice satisfies the following properties.*

(34) $$\begin{array}{cc} Extensivity & X⊥\;⊥Y|Z\;\mathrm{implies}\;X⊥\;⊥Y⊎Z|Z\;, \end{array}$$

(35) $$\begin{array}{cc} Transitivity & \mathrm{If}\;X⊥\;⊥Y|W\;\mathrm{and}\;X⊥\;⊥Z|Y⊎W,\;\mathrm{then}\;X⊥\;⊥Z|W\;. \end{array}$$

**Remark** **3.** *Property (34), which we call extensivity, was called normality by Paolini [26].*

**Proof.**  To prove the extensivity (34), assume that X⊥⊥Y∣Z, which is equivalent to X⊥⊥Y∣Z⊎Z. The monotonicity (31) gives X⊥⊥Z∣Z. The conclusion X⊥⊥Y⊎Z∣Z is obtained by the chain rule (33).

To prove the transitivity (35), assume that X⊥⊥Y∣W and X⊥⊥Z∣Y⊎W. The chain rule (33) applied twice gives X⊥⊥Y⊎Z∣W and X⊥⊥Z∣W. □

In a set of random variables, we note that if *Y* is independent of *Y* given *X*, then *Y* is a function of *X* almost surely. If Y⊥⊥Y∣X, we write $X\to_{⊥\;⊥}Y$.

**Theorem** **3.** *If $\left(L,\cap ,⊎\right)$ is a lattice with a separoid relation $\left(\cdot ⊥\;⊥\cdot |\cdot \right)$, then the relation $\to_{⊥\;⊥}$ satisfies Armstrong’s axioms. The relation $\left(\cdot ⊥\;⊥\cdot |\cdot \right)$ restricted to the lattice of closed lattice elements is separoid.*

**Proof.**  The extensivity (18) of $\to_{⊥\;⊥}$ follows directly from the extensivity (34) of ⊥⊥.

The monotonicity (19) follows directly from the monotonicity (31).

To prove the transitivity of $\to_{⊥\;⊥}$, assume that $X\to_{⊥\;⊥}Y$ and $Y\to_{⊥\;⊥}Z$. The monotonicity (31) implies that Z⊥⊥X∣Z⊎Y, which by the chain rule (33), implies Z⊥⊥Z⊎X∣Y. By the chain rule (33), we have Z⊥⊥Z∣Y⊎X. The monotonicity (31) also gives Z⊥⊥Y∣Y⊎X, which together with $X\to_{⊥\;⊥}Y$ implies that Z⊥⊥Y∣X by transitivity (35). The transitivity (35) then implies Z⊥⊥Z∣X.

To prove that the relation $\left(\cdot ⊥\;⊥\cdot |\cdot \right)$ restricted to the lattice of closed lattice elements is separoid, one just has to prove that X⊥⊥Y∣Z if and only if $X⊥\;⊥cl_{⊥\;⊥}\left(Y\right)|Z$ if and only if $X⊥\;⊥Y|cl_{⊥\;⊥}\left(Z\right)$. This follows from Armstrong’s results.

The significance of this theorem is that if we start with a separoid relation on a lattice, then this separoid relation is also a separoid when restricted to elements that are closed under the relation $\to_{⊥\;⊥}$.

**Theorem** **4.** *Any finite lattice can be represented as a closure system of a separoid relation defined on a powerset.*

**Proof.**  For any finite lattice L, one identifies the elements with subgroups of a group *G*. If the group *G* is assigned a uniform distribution, then the variable corresponding to a subgroup will also have a uniform distribution. With this distribution, a variable is independent of itself given another variable if and only if the other variable determines the first variable. Therefore, statistical independence with respect to the uniform distribution on *G* gives a separoid relation for which the closure is the original lattice. □

Assume that *X* and *Y* are $\to_{h}$ closed. Then:

(36) $$\begin{array}{cc} h\left(cl_{\to_{h}}\left(X⊎Y\right)\right)+h\left(X\cap Y\right) & =h\left(X⊎Y\right)+h\left(X\cap Y\right)\\ & \leq h\left(X\right)+h\left(Y\right). \end{array}$$

Therefore, *h* restricted to the $\to_{h}$ closed elements is polymatroid. We may summarize these observations in the following proposition.

**Proposition** **5.** *If h is a polymatroid function defined on the lattice $\left(L,\subseteq \right)$, then the relation $\to_{h}$ satisfies Armstrong’s axioms. The function h restricted to the lattice of $\to_{h}$ closed elements is polymatroid.*

We recall that a pair of point $\left(Y,Z\right)$ is said to be a modular pair, and we write YMZ if Y∩Z⊆X⊆Z implies that:

(37) $$\left(X⊎Y\right)\cap Z=X\;.$$

If all pairs are modular, we say that the lattice is modular, and we have:

(38) $$\begin{array}{cc} The\;modular\;law & X⊎\left(Y\cap Z\right)=\left(X⊎Y\right)\cap Z\;.\;\; \end{array}$$

when X⊆Z.

**Proposition** **6.** *If $\left(\cdot ⊥\;⊥\cdot |\cdot \right)$ is a separoid relation on a lattice and:*

(39) Y⊥⊥Z∣Y∩Z

*then YMZ in the lattice of closed elements. In particular, if h is a polymatroid function on a lattice and:*

(40) h(Y)+h(Z)=h(Y∩Z)+h(Y⊎Z),

*then YMZ in the lattice of closed elements.*

**Proof.**  If Y∩Z⊆X⊆Z, then we have the following sequence of implications.

(41) $$\begin{array}{c} Y⊥\;⊥Z|Y\cap Z \end{array}$$

(42) $$\begin{array}{c} Y⊥\;⊥X⊎Z|Y\cap Z \end{array}$$

(43) $$\begin{array}{c} Y⊥\;⊥Z|X⊎\left(Y\cap Z\right) \end{array}$$

(44) $$\begin{array}{c} Y⊥\;⊥Z|X \end{array}$$

(45) $$\begin{array}{c} X⊎Y⊥\;⊥Z|X \end{array}$$

(46) $$\begin{array}{c} \left(X⊎Y\right)\cap Z⊥\;⊥\left(X⊎Y\right)\cap Z|X \end{array}$$

Hence,

(47) $$X\to_{⊥\;⊥}\left(X⊎Y\right)\cap Zandcl\left(X\right)=cl\left(\left(X⊎Y\right)\cap Z\right).$$

 □

If ⊥⊥ is separoid, then according to the extensivity (34), the relation X⊥⊥Y∣Z implies:

(48) X⊎Z⊥⊥Y⊎Z∣Z

so that $Z{⊇}_{⊥\;⊥}\left(X⊎Z\right)\cap \left(Y⊎Z\right){⊇}_{⊥\;⊥}Z.$ Following Dawid [24], we define the relation $X{⊥\;⊥}_{M}Y|Z$ by:

(49) $$Z=\left(X⊎Z\right)\cap \left(Y⊎Z\right).$$

**Theorem** **5.** *If a polymatroid function h on a lattice is modular, then the lattice of $\to_{h}$ closed elements is modular. If the lattice is modular, then $X{⊥\;⊥}_{h}Y|Z$ if and only if $X{⊥\;⊥}_{M}Y|Z$ in the lattice of closed elements.*

**Proof.**  If the function *h* is modular, then all pairs of elements are modular in the lattice of *h*-closed elements, so the lattice of closed elements is modular. In a modular lattice:

(50) $$I_{h}\left(X,Y|Z\right)=h\left(\left(X⊎Z\right)\cap \left(Y⊎Z\right)\right)-h\left(Z\right)$$

so that $X{⊥\;⊥}_{h}Y|Z$ holds when $Z\to_{h}\left(X⊎Z\right)\cap \left(Y⊎Z\right).$ □

The following result appears in [24] with a longer proof.

**Corollary** **2.** *For a lattice, the relation $X{⊥\;⊥}_{M}Y|Z$ is separoid if and only if the lattice is modular.*

**Proof.**  Assume that the lattice is modular. Then, the ranking function *r* is modular, and $X\to_{r}Y$ if and only if X⊇Y. Therefore, $X{⊥\;⊥}_{M}Y|Z$ is equivalent to the separoid relation $I_{r}\left(X,Y|Z\right)=0$.

Assume that the relation ${⊥\;⊥}_{M}$ is separoid. Since $X{⊥\;⊥}_{M}Y|X\cap Y$, we have that XMY. Since all pairs are modular, the lattice is modular.

## 4. Entropy in Functional Dependence Lattices

Let L denote a lattice with maximal element *m*. Let $\Gamma \left(L\right)$ denote the set of polymatroid functions on L. The set $\Gamma \left(L\right)$ is polyhedral, and often, we may normalize the polymatroid functions by replacing $h\left(\cdot \right)$ by $h\left(\cdot \right)/h\left(m\right)$. In this way, we obtain a polytope that we will denote $\Gamma_{1}\left(L\right)$.

**Definition** **2.** *A function $h\in \Gamma \left(L\right)$ is said to be entropic if there exists a function f from L into a set of random variables such that $h\left(X\right)=H\left(f\left(X\right)\right)$ for any element X in the lattice.*

Let $\Gamma_{1}^{*}\left(L\right)$ denote the set of normalized entropic functions on L, and let ${\bar{\Gamma }}_{1}^{*}\left(L\right)$ denote the closure of $\Gamma_{1}^{*}\left(L\right)$.

**Definition** **3.** A lattice is said to be a Shannon lattice if any polymatroid function can be realized approximately by random variables, i.e., $\Gamma_{1}\left(L\right)={\bar{\Gamma }}_{1}^{*}\left(L\right).$

One may then check whether a lattice is a Shannon lattice by checking that the extreme polymatroid functions are entropic or can be approximated by entropic functions.

**Example** **4.** *Let G denote a finite group. For any subgroup $\tilde{G}\subseteq G$, we associate the variable $X_{\tilde{G}}$ that maps an element g∈G into the left coset $g\tilde{G}.$ The number of possible values of $X_{\tilde{G}}$ is $\left|G:\tilde{G}\right|=\frac{\left|G\right|}{\left|\tilde{G}\right|}$. Assume that the subgroups are given a functional dependence structure where a variable X is given by a function $A\to_{B}$. If A has n elements, then the groups of permutations G have n! elements. The subgroup that leaves X invariant has:*

(51) $$\Pi_{b\in B}\left(n\cdot P\left(X=b\right)\right)!$$

*element. Therefore:*

(52) $$\begin{array}{cc} \ln\left(\left|G:G_{X}\right|\right) & =\ln\left(\frac{n}{\Pi_{b\in B}\left(n\cdot P\left(X=b\right)\right)!}\right)\\ & \approx -n\cdot \sum_{b\in B}P\left(X=b\right)\ln\left(P\left(X=b\right)\right)\\ & =n\cdot H\left(X\right). \end{array}$$

If U is the uniform distribution on the finite group G, then the distribution of $X_{\tilde{G}}$ is uniform, and the entropy is $H\left(X_{\tilde{G}}\right)=\ln\left(\left|G\right|\right)-\ln\left(\left|\tilde{G}\right|\right).$ It has been proven that the set of entropic functions generated form a convex cone. Therefore, the normalized polymatroid functions generated by groups has ${\bar{\Gamma }}_{1}^{*}\left(L\right)$ as closure [4].

From Definition 3, we immediately get the following result.

**Proposition** **7.** *If L is a Shannon lattice and M is a subset that is a ∩-semi-lattice, then M is a Shannon lattice. In particular, all sub-lattices of a Shannon lattice are Shannon lattices.*

**Proof.**  Assume that L is a Shannon lattice and that *M* is a sub-lattice. Let h:M→R denote a polymatroid function. For ℓ∈L, let $\tilde{\ell }$ denote the m∈M that minimize $h\left(m\right)$ under the constraint that m⊇ℓ. Define the function $\tilde{h}\left(\ell \right)=h\left(\tilde{\ell }\right).$ Now, $\tilde{h}$ is an extension of *h*, and with this definition, $\tilde{h}$ is non-negative and increasing. For x,y∈L, we have:

(53) $$\begin{array}{cc} \tilde{h}\left(X\right)+\tilde{h}\left(Y\right) & =h\left(\tilde{X}\right)+h\left(\tilde{Y}\right)\\ & \geq h\left(\tilde{X}⊎\tilde{Y}\right)+h\left(\tilde{X}\cap \tilde{Y}\right)\\ & \geq \tilde{h}\left(X⊎Y\right)+\tilde{h}\left(X\cap Y\right) \end{array}$$

because $\tilde{X}⊎\tilde{Y}\geq X⊎Y$ and $\tilde{X}\cap \tilde{Y}\geq X\cap Y.$ Hence, $\tilde{h}$ is submodular. By the assumption, $\tilde{h}$ is entropic, so the restriction of $\tilde{h}$ to *M* is also entropic. □

With these results it hand, we can start hunting for non-Shannon lattices. We take a lattice that may or may not be a Shannon lattice. We find the extreme normalized polymatroid functions. These extreme polymatroid functions can be found either by hand or by using some suitable software that can find extreme points of a convex polytope specified by a finite set of inequalities. For instance, the R program with package rcdd can find all extreme points of a polytope. For each extreme point, we determine the lattice of closed elements using Proposition 5. These lattices of closed sets will often have a much simpler structure than the original lattice, and the goal is to check if these lattices are Shannon lattices or not. It turns out that there are quite a few of these reduced lattices, and they could be considered as the building blocks for larger lattices.

We recall that an element *i* is ⊎-irreducible if i=X⊎Y implies that i=X or i=Y. An ∩-irreducible element is defined similarly. An element is double irreducible if it is both ⊎-irreducible and ∩-irreducible. The lattice denoted $M_{n}$ is a modular lattice with a smallest element, a largest element and *n* double irreducible elements arranged in-between.

**Theorem** **6.** *For any n, the lattice $M_{n}$ is a Shannon lattice.*

**Proof.**  The proof is essentially the same as the solution to the cryptographic problem stated at the beginning of Section 2. The idea is that one should look for groups with a subgroup lattice $M_{n}$ and then check that the subgroups of such group have the right cardinality.

Let the values in the double irreducible elements be denoted $h_{1},h_{2},\cdots ,h_{n}$. If n=1, the extreme polymatroid functions are $h_{1}=0$ and $h_{1}=1$, and these points are obviously entropic. If n=2, the extreme points are $\left(h_{1},h_{2}\right)=\left(0,1\right)$, $\left(h_{1},h_{2}\right)=\left(1,0\right)$ and $\left(h_{1},h_{2}\right)=\left(1,1\right),$ which are all entropic.

Assume n≥3. Then, the values should satisfy the inequalities:

(54) $$\begin{array}{cc} 0\leq h_{i} & \leq 1\;, \end{array}$$

(55) $$\begin{array}{cc} h_{i}+h_{j} & \geq 1\;. \end{array}$$

If $\left(h_{1},h_{2},\cdots ,h_{n}\right)$ is an extreme point, then each variable should satisfy one of the inequalities with equality. Assume $h_{i}=0.$ Then, sub-modularity implies that $h_{j}=1$ for j≠i. The extreme point $\left(1,1,\cdots ,1,0,1,\cdots ,1\right)$ is obviously entropic. If $h_{i}=1$, this gives no further constraint on the other values, so it corresponds to an extreme point on a lattice with one less variable. Finally, assume that $h_{i}+h_{j}=1$ for all i,j. Then, hi=12 for all i. □

**Corollary** **3.** *Any polymatroid function that only takes the values 0,12 and* 1 *is entropic.*

**Proof.**  Assume that the polymatroid function *h* only takes the values 0,12, and 1. Then, *h* defines a separoid relation, and the closed elements form a lattice isomorphic to $M_{n}$ for some integer *n*. The function *h* is entropic on $M_{n}$, so *h* is also entropic on the original lattice. □

**Lemma** **1.** *If h is submodular and increasing on ∩-irreducible elements, then h is increasing.*

**Proof.**  Assume that *h* is submodular and increasing on ∩-irreducible elements. We have to prove that if X⊇Z, then $h\left(X\right)\geq h\left(Z\right).$ In order to obtain a contradiction, assume that *Z* is a maximal element such that there exist an element *X* such that X⊇Z, but $h\left(X\right)<h\left(Z\right).$ We may assume that *X* cover *Z*. Since *h* is increasing at ∩-irreducible elements, *Z* cannot be ∩-irreducible. Therefore, there exists a maximal element *b* such that Y⊇Z, but Y⊉X. Since *X* cover *Z*, we have X∩Y=Z. According to the assumptions, $h\left(X\right)+h\left(Y\right)\geq h\left(X⊎Y\right)+h\left(X\cap Y\right)$ and $h\left(X⊎Y\right)\geq h\left(Y\right)$ because *Z* is a maximal element that violates that *h* is increasing. Therefore, $h\left(X\right)\geq h\left(X\cap Y\right)=h\left(Z\right).$ □

**Theorem** **7.** *Any lattice with seven or fewer elements is a Shannon lattice.*

**Proof.**  Up to isomorphism, there only exist finitely many lattices with seven elements or less. These are listed in the Appendix B. Each of these lattices has finitely many extreme polymatroid functions. These extreme polymatroid functions can be found by hand or by using the R program with package rcdd. All the extreme polymatroid functions on these lattices can be represented by a trivial lattice, or by the two-element chain **2**, or by $M_{5}$, or by $M_{6}$, or by $M_{7}$. All these lattices are representable, and thereby, they are Shannon lattices. □

The number of lattices grows quite fast with the number of elements, and the number of elements is not the best way of comparing lattices.

**Figure 2:**
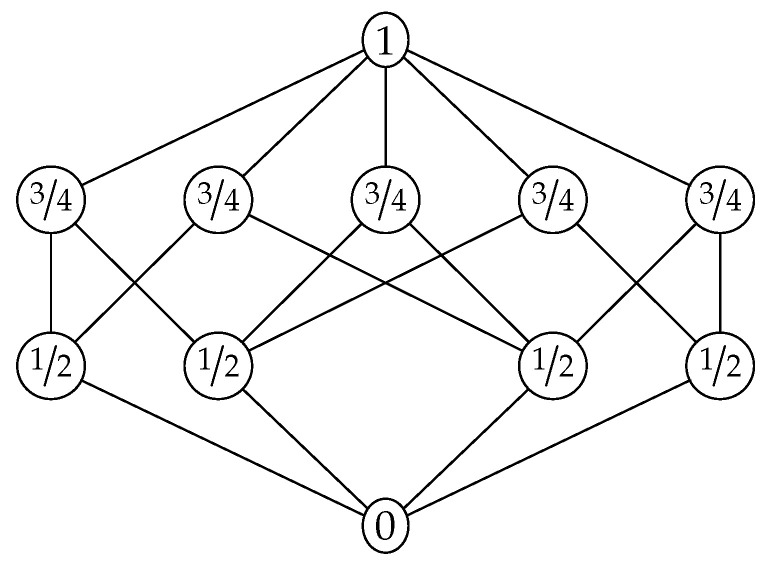
The Matúš lattice with a non-entropic polymatroid function. This lattice is named in honor of František Matúš, who passed away shortly before the submission of this manuscript.

The Boolean lattice with four atoms is the smallest non-Shannon Boolean algebra. Nevertheless, there are smaller non-Shannon lattices. Figure 2 illustrates the Matúš lattice, which is a lattice with just 11 elements that violates Inequality (1). This corresponds to the fact that the lattice in Figure 2 is not equivalent to a lattice of subgroups of a finite group. The lattices that are equivalent to lattices of subgroups of finite groups have been characterized [30], but the characterization is too complicated to describe here. Using the ideas from [31], one can prove that the Matúš lattice in Figure 2 has infinitely many non-Shannon inequalities. Therefore, any lattice that contains the Matúš lattice as a ∩-semilattice also has infinitely many non-Shannon inequalities.

**Conjecture** **1.** *A lattice is a Shannon lattice if and only if the lattice does not contain the Matúš lattice as a ∩-semilattice.*

The result of Matúš has recently found a parallel in matroid theory. An infinite set of inequalities is needed in order to characterize presentable matroids [32,33,34].

## 5. The Skeleton of a Lattice

In this section, we will develop a cutting-and-gluing technique that can be used to handle many lattices, but it is especially useful for planar lattices. We present the notion of tolerance. Further details about this concept can be found in the literature [5,35].

**Definition** **4.** *A symmetric and reflexive relation *Θ* on a lattice is called a tolerance relation if $X_{1}\Theta X_{2}$ and $Y_{1}\Theta Y_{2}$ imply:*

(56) $$\left(X_{1}\cap X_{2}\right)\Theta \left(Y_{1}\cap Y_{2}\right)\;$$

*and*

(57) $$\left(X_{1}⊎X_{2}\right)\Theta \left(Y_{1}⊎Y_{2}\right).$$

If Θ is a tolerance relation, then for any *X*, the set $\left\{Y\in L|X\Theta Y\right\}$ is an interval in the lattice. These intervals are called the blocks of Θ, and the blocks will be denoted ${\left[X\right]}_{\Theta }.$ For a tolerance relation, the blocks may be considered as elements of the factor L/Θ, and this factor has a natural structure as a lattice. Congruence relations are special cases of tolerance relations, but in general, the blocks of a tolerance relation may overlap. We note that if the intersection of two blocks is non-empty, then the intersection is a sublattice. If X∈L/Θ, then $L_{X}$ will denote the block in L determined by X. We defined a glued tolerance relation as a tolerance relation where *X* cover *Y* in L/Θ, implying that $L_{X}\cap L_{Y}\neq \emptyset .$

**Figure 3:**
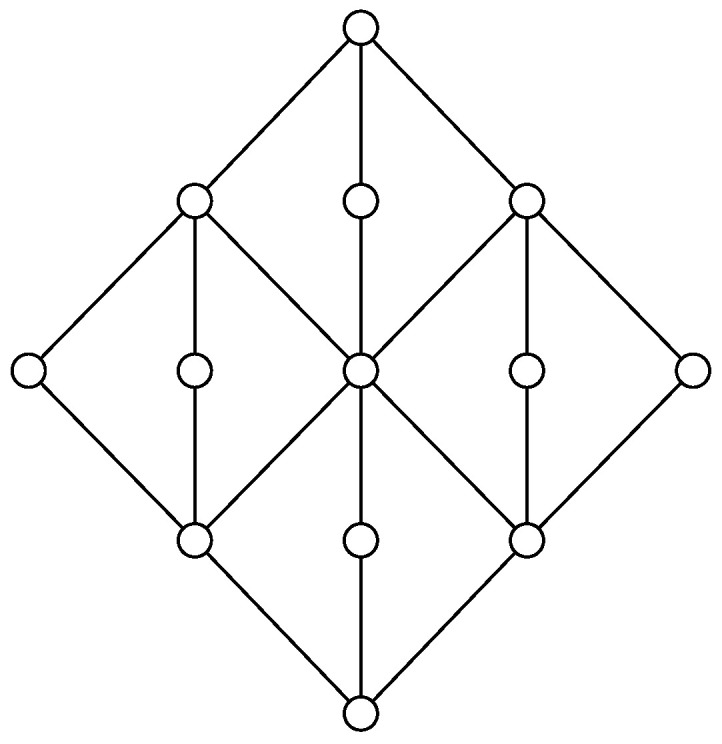
A planar modular lattice.

**Figure 4:**
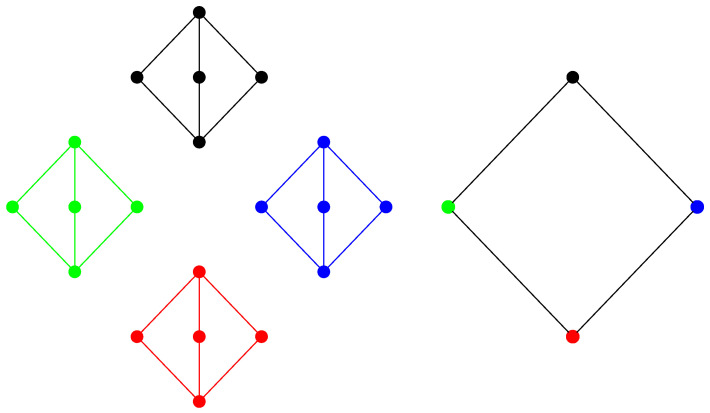
The skeleton of the lattice in the previous figure. It consist of four blocks glued together by the factor lattice illustrated to the right.

A tolerance relation can be identified with a subset of L×L, so tolerance relations are ordered by subset ordering. The trivial tolerance relation is the one where xΘy holds for all x,y∈L, and this tolerance relation is the greatest tolerance relation. A glued tolerance relation contains any covering pair, and glued tolerance relations are characterized by this property. Therefore, the intersection of two glued tolerance relations is a glued tolerance relation. Therefore, the set of glued tolerance relations forms a lattice. The smallest glued tolerance relation is denoted $\Sigma \left(L\right)$ and is called the skeleton of the lattice. An example of a planar modular lattice is given in Figure 3 and the skeeton is given in Figure 4.

**Lemma** **2.** *Let L be a lattice with an increasing function h. If the function h satisfies:*

(58) $$h\left(X\right)+h\left(Y\right)\geq h\left(X\cap Y\right)+h\left(X⊎Y\right)$$

*for all X,Y where X∩Y is covered by X and Y, then the function h is submodular on L.*

**Proof.**  First, we prove that if the function *h* satisfies:

(59) $$h\left(X\right)+h\left(Y\right)\geq h\left(X\cap Y\right)+h\left(X⊎Y\right)$$

for all X,Y where X∩Y is covered by *X*, then the function *h* is submodular on L.

Let *A* and *A* denote two lattice elements. Define sequences $X_{1}\subseteq X_{2}\cdots \subseteq X_{n}=A$ and $Y_{1}\subseteq Y_{2}\cdots \subseteq Y_{n}=A⊎B$ by first defining $X_{1}=A\cap B$ and $Y_{1}=B.$ Assume that $X_{1}$ is an element that covers A∩B and such that $X_{1}\leq A.$ Let $X_{i+1}\subseteq A$ be a cover of $A\cap Y_{i}$, and let $Y_{i+1}=X_{i+1}⊎Y_{i}.$ Then:

(60) $$h\left(X_{i+1}\right)+h\left(Y_{i}\right)\geq h\left(Y_{i+1}\right)+h\left(X_{i+1}\cap Y_{i}\right).$$

Adding all these inequalities leads to:

(61) $$h\left(A\right)+h\left(B\right)\geq h\left(A⊎B\right)+h\left(A\cap B\right)+\sum_{i=0}^{n-1}\left(h\left(X_{i+1}\cap Y_{i}\right)-h\left(X_{i}\right)\right)$$

and the inequality is obtained because *h* is increasing to that $h\left(X_{i+1}\cap Y_{i}\right)-h\left(X_{i}\right)\geq 0$ and because $X_{i+1}\cap Y_{i}⊇X_{i}$ by construction of the sequences.

To see that, we just need to check submodularity when *B* covers A∩B proven in the same way. □

**Proposition** **8.** *Let L be a lattice with a tolerance relations *Θ*, and let h:L→R denote some function. Then, h is polymatroid if and only if the restriction of h to any block $L_{x}$ is polymatroid.*

If *h* is entropic, then the restriction to each block is entropic. Characterizing the blocks of a lattice has been done for certain classes of lattices, but here, we shall only mention a single result.

**Theorem** **8** ([36])**.** *The blocks of a modular lattice are the maximal atomistic intervals.*

In particular, the skeleton of a modular lattice consists of blocks that are geometric lattices.

## 6. Results for Planar Lattices

In this section, we will restrict our attention to planar lattices. There are several reasons for this restriction. First of all, any poset with a planar Hasse diagram is a lattice if and only if it has a least element and a greatest element [37]. As a consequence, any ∩-semilattice of a planar lattice is also a planar lattice. Certain cut-and-glue techniques are also very efficient for planar lattices. Finally, both planar distributive lattices and planar modular lattices have nice representations that will play a central role in our proofs.

**Theorem** **9.** *Let h denote a polymatroid function on a planar lattice L. Then, h has an entropic representation if and only if the restriction to each block of $\Sigma \left(L\right)$ has an entropic representation.*

**Proof.**  The proof is via induction over the number of elements in the lattice. For a trivial lattice, there is nothing to prove. Assume that the theorem has been proven for all lattices with fewer elements than the number of elements of L. Assume that *h* is a polymatroid. Since the lattice is planar, it has a left boundary chain $\emptyset \subset L_{1}\subset L_{2}\cdots \subset L_{m}$ and a right boundary chain $\emptyset \subset R_{1}\subset R_{2}\cdots \subset R_{n}$ where $L_{m}=R_{n}$ is the maximal element of L. Let $R_{k}$ be the minimal element of the right boundary chain such that $L_{1}\subseteq R_{k}.$ We note that $R_{k}=L_{1}⊎R_{k-1}.$ Let $L_{j}$ denote the largest element in the left boundary chain such that $L_{j}\subseteq R_{k}.$ Then, there is a chain from $L_{j}$ to $R_{k}$, and we have a glued tolerance relation with two blocks $L_{0}=\left\{X\in L|X\subseteq R_{k}\right\}$ and $L_{1}=\left\{X\in L|X⊇L_{j}\right\}$ and with the two element chain lattice **2** as the factor lattice. These two blocks are glued together along a chain $L_{j}=y_{1}\subset y_{2}\subset \cdots \subset y_{t}=R_{k}$ that $L_{1}$ and $L_{0}$ share. There are two cases: either $R_{k}\subset R_{n}$ or $R_{k}=R_{n}.$

Assume that $R_{k}\subset R_{n}.$ Then, the glued tolerance relation is non-trivial. Since *h* restricted to $\left\{X\in L|X⊇L_{j}\right\}$ and $\left\{X\in L|X\subseteq R_{k}\right\}$ are probabilistically representable, we may without loss of generality assume that there exist two groups $G_{1}$ and $G_{0}$ such that to $X\in L_{i}$, there is a subgroup $G_{i}\left(X\right)\subseteq G_{i}$ such that $h\left(X\right)=\ln\left|G_{i}:G_{i}\left(X\right)\right|.$ We associate the variable $X_{G_{i}\left(X\right)}$ that maps an element g∈G into the left coset $gG_{i}\left(X\right).$ The goal is to find a joint distribution to a set of variables associated with each X∈L. We note that all variables in $L_{0}$ are functions of $r_{k}$, so if we map $X_{G_{1}\left(r_{k}\right)}$ into $X_{G_{0}\left(r_{k}\right)}$, all other variables in $L_{2}$ are determined. In particular, the chain $y_{1}\subset y_{2}\subset \cdots y_{t}$ is determined by $r_{k}=y_{t}.$ The sequences $X_{G_{1}\left(y_{i}\right)}$ are mapped into the sequence $X_{G_{0}\left(y_{i}\right)}$ recursively, starting with mapping $X_{G_{1}\left(y_{1}\right)}$ into $X_{G_{0}\left(y_{1}\right)}.$ This is possible since $X_{G_{1}\left(y_{1}\right)}$ and $X_{G_{2}\left(y_{1}\right)}$ are uniform distributions on sets of the same size. Now, there are equally many values of $X_{G_{1}\left(y_{2}\right)}$ and $X_{G_{0}\left(y_{2}\right)}$ that map into the same values of $X_{G_{1}\left(y_{1}\right)}$ and $X_{G_{0}\left(y_{1}\right)}$, so the the values of $X_{G_{1}\left(y_{2}\right)}$ and $X_{G_{0}\left(y_{2}\right)}$ can be mapped into each other. We continue like that until all the random variables along the chain $y_{1}\subset y_{2}\subset \cdots y_{t}$ have been identified.

If $r_{k}=r_{n}$, then we make a similar construction with the role of the left chain and the right chain reversed. If this leads to a non-trivial glued tolerance relation, we glue representations together as we did above.

If both the left chain and the right chain lead to trivial glued tolerance relations, then $L_{1}⊎r_{1}$ is the maximal element of L, and the whole lattice consists of a single block in $\Sigma \left(L\right).$ In this case, the content of the theorem is trivial. □

**Theorem** **10.** *All planar modular lattices are Shannon lattices.*

**Proof.**  Without loss of generality, we may assume that the lattice consists of just one block for the tolerance relation $\Sigma \left(L\right).$ A modular block is atomistic, but if a modular planar lattice is atomistic, it is equivalent to the trivial lattice or to the lattice **2**, or to the lattice 2×2, or to one of the lattices $M_{n}.$ □

**Figure 5:**
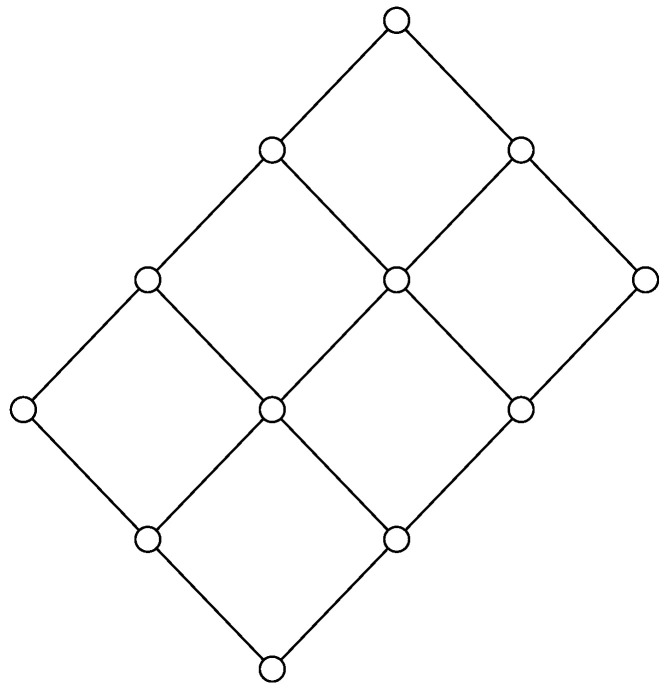
A product of two chains.

Our construction actually tells us more. If the lattice is distributive, it is glued together with blocks that are either equivalent to **2** or to the lattice 2×2. Therefore, the lattice is a sublattice of a product of two chains, as illustrated in Figure 5. This result was first proven by Dilworth [38]. Other characterizations of planar distributive lattices can be found in the literature [39]. Since the extreme polymatroid functions on the lattices **2** and the lattice 2×2 only take the values zero and one, the same is true for any planar distributive lattice.

**Figure 6:**
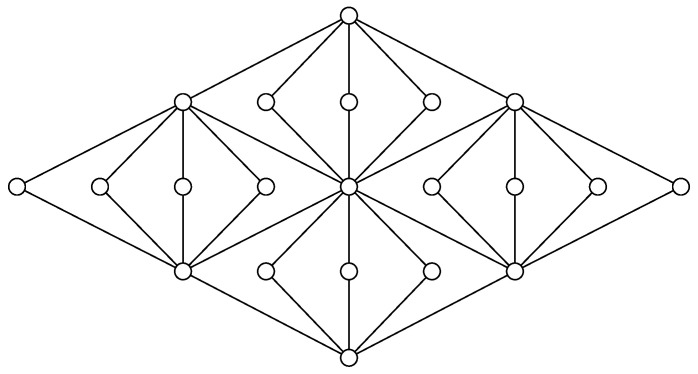
A planar modular lattice.

A modular planar lattice will also contain blocks of the type $M_{n}$. Therefore, a modular planar lattice can be obtained from a distributive planar lattice by adding double irreducible elements [40], as illustrated in Figure 6.

Since $M_{n}$ has extreme polymatroid functions that take the values 0, 12 and 1, the extreme functions are modular. Gluing such modular functions together leads to extreme polymatroid functions that are modular. Therefore, all extreme polymatroid functions on a planar modular lattice can be represented by a planar modular lattice with a modular function. Therefore, the independence structure is given by $\left(X⊥\;⊥Y|Z\right)$ when $Z=\left(X⊎Z\right)\cap \left(Y⊎Z\right).$

**Figure 7:**
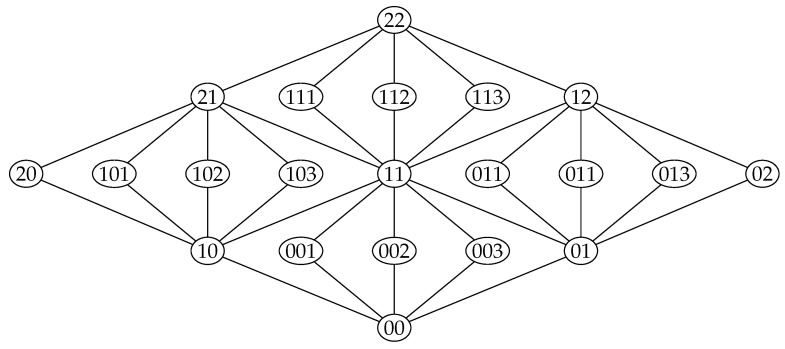
A planar modular lattice with indexing of the elements.

The extreme polymatroid functions on a planar modular lattice can be represented as follows. Let $X_{1},X_{2},\cdots X_{m},Y_{1},Y_{2},\cdots ,Y_{n}$ denote independent random variables uniformly distributed over $Z_{p}$ for some large value of p. Let $Z_{ij}$ denote the random variable:

(62) $$\underset{\ell \leq i}{⊎}X_{\ell }⊎\underset{\ell \leq j}{⊎}Y_{\ell }.$$

and let $Z_{ijk}$ denote the random variable:

(63) $$\underset{\ell \leq i}{⊎}X_{\ell }⊎\underset{\ell \leq j}{⊎}Y_{\ell }⊎\left(X_{i+1}+k\cdot Y_{j+1}\right)$$

for k>0. The way to index the variables can be seen in Figure 7. Then, the entropy is proportional to the ranking function. A polymatroid function *h* that has a representation given by an Abelian group satisfies the Ingleton inequalities [41], i.e., inequalities of the form:

(64) $$\begin{array}{c} h(X)+h(Y)+h(X⊎Y⊎V)+h(X⊎Y⊎W)+h(V⊎W)\leq \\ \;h(X⊎Y)+h(X⊎V)+h(X⊎W)+h(Y⊎V)+h(Y⊎W). \end{array}$$

Therefore, the Shannon inequalities imply the Ingleton inequalities as long as the polymatroid function is defined on a planar modular lattice. Paajanen [42] has proven that under some conditions, the entropy function of a nilpotent *p*-group can be represented by an Abelian group. The core of the proof was that the subgroup lattice of a nilpotent *p*-group is also the subgroup lattice of an Abelian group. Many of these lattices are planar, and in these cases, the results by Paajanen follow from our results on planar graphs.

## 7. Discussion

In this paper, we have proven that the three basic Shannon inequalities are sufficient for certain lattices. It would be a major step forward if one could make a complete characterization of lattices without non-Shannon inequalities, but this may be too ambitious. In order to obtain results, one may have to restrict to certain classes of lattices like general modular lattices or geometric lattices. For handling such lattices, one would have to develop new techniques that may also be of wider interest.

Lattices seem to fall into two types. For one type, one does not have non-Shannon inequalities. For the other type, there are infinitely many non-Shannon inequalities. We do not know of any lattice with non-Shannon inequalities where the entropic functions are characterized by finitely many inequalities. Apparently, the complexity increases from three basic inequalities to infinitely many inequalities, and this transition seems to happen due to the Matúš lattice. Similarly matroids in general have no finite characterization, and conditional independence does not have a finite characterization. It appear to be the case that the leap from low complexity to infinite complexity happens for the same reason and seems to be related to the structure of the Matús lattice. In this paper, we have provided some basic results and a common terminology that should be useful for further exploration of this research area.

Bayesian networks and similar graphical models have not been discussed in the present paper. Nevertheless, Bayesian networks are closely related to functional dependencies, so important properties of Bayesian networks can be translated into lattice language. This will be the topic of a separate publication [43], but some preliminary results have already been published [7].

We have seen how a separoid relation generates a notion of functional dependence. For modular lattices, we have also seen that the lattice structure generates a separoid relation. It is an open question to what extent general lattices are born with a canonical notion of conditional independence that can be formalized in terms of separoids. For functional dependencies corresponding to Bayesian networks, this question has been studied in detail [16], but more general results related to these questions would be of great importance to our understanding of concepts related to cause and effect.

## Appendix A. Augmentation

In database theory extensivity (18) is replaced by the following property:.

(A1) $$\begin{array}{cc} Augmentation & X\to Y\;\mathrm{implies}\;X⊎Z\to Y⊎Z\;. \end{array}$$

If Z=X, the augmentation (A1) reduces to the extensivity (18). In a finite lattice, the extensivity (18) together with the other Armstrong axioms imply the augmentation (A1). To see this, first, we observe that in a finite lattice extensivity (18), monotonicity (19) and transitivity (20) imply that X→Y is equivalent to $cl\left(X\right)⊇Y$. The monotonicity (16) gives cl(X⊎Z)≥Y. Using the monotonicity (16) and extensivity (15), we also get cl(X⊎Z)≥cl(Z)≥Z. Combining these two inequalities gives cl(X⊎Z)≥Y⊎Z, as desired.

The condition in Theorem 1 that the lattice is finite can be relaxed to the ascending chain condition, because this is essentially what is used to conclude that the chain (22) must stop. The observation that augmentation can be relaxed to extensivity could be used to simplify some algorithms for database normalization.

## Appendix B. Lattices of Size 1–7

Here, we give a complete list of the Hasse diagrams of lattices with seven or fewer elements.

### Appendix B.1. Lattice of Size 1

The trivial lattice is the only lattice of Size 1.

### Appendix B.2. Lattice of Size 2

The two element chain **2** is the only lattice of Size 2.

### Appendix B.3. Lattices of Size 3

The three element chain **3** is only one lattice of Size 3, and and it is distributive. The extreme polymatroid functions can be represented by the lattice **2**.

### Appendix B.4. Lattices of Size 4

There are two lattices of Size 4, and they are both distributive. Their extreme polymatroid functions can be represented by the lattice **2**.

### Appendix B.5. Lattices of Size 5

The lattice $M_{5}$ is modular, but not distributive. It has a a new non-trivial polymatroid function as the extreme point. The other extreme points can be represented by $M_{3}$ and the lattice **2**.

### Appendix B.6. Lattices of Size 6

The lattice $M_{6}$ has a new non-trivial polymatroid extreme point. The other extreme points can be represented by $M_{3}$ and the lattice: **2**.

The next five lattices have extreme points that can be represented by $M_{5}$ or the lattice **2**. The first two lattices are modular, but not distributive. The next three are not modular.

The extreme points of the last nine lattices are all represented by the lattice **2**. The first four are not modular.

The last five are distributive.

### Appendix B.7. Lattices of Size 7

The lattice $M_{5}$ has a new polymatroid extreme point. The other extreme points can be represented by $M_{4}$, $M_{3}$ and the lattice **2**.

The next seven lattices have extreme points that can be represented by $M_{4}$, $M_{3}$ or the lattice **2**. The first two lattices are modular. The last five lattices are not modular.

The following lattices have extreme polymatroid functions that can be represented by $M_{3}$ or the lattice **2**. The first five lattices are modular.

The next lattices are not modular.

The last 22 lattices of Size 7 only have trivial extreme points. The first 14 lattices are not modular.

The last eight lattices are distributive.

## References

[1] Zhang Z., Yeung R.W. (1998). On characterization of entropy function via information inequalities. IEEE Trans. Inform. Theory.

[2] Shannon C.E. (1948). A mathematical theory of communication. Bell Syst. Tech. J..

[3] McGill W. (1954). Multivariate information transmission. Psychometrika.

[4] Yeung R.W. (2002). A First Course in Information Theory.

[5] Stern M. (1999). Semimodular Lattices. Theory and Applications.

[6] Chan T.H., Yeung R.W. (2002). On a Relation between Information Inequalities and Group Theory. IEEE Trans. Inform. Theory.

[7] Harremoës P., Rissanen J., Myllymäki P., Teemu Roos I.T., Yamanishi K. (2011). Functional Dependences and Bayesian Networks. Proceedings of the WITMSE 2011.

[8] Harremoës P. Lattices with non-Shannon inequalities. Proceedings of the 2015 IEEE International Symposium on Information Theory.

[9] Lee T.T. (1983). An algebraic theory of relational databases. Bell Syst. Tech. J..

[10] Demetrovics J., Libkin L., Muchnik I.B. (1989). Functional dependencies and the semilattice of closed classes. Proceedings of the 2nd Symposium on Mathematical Fundamentals of Database Systems (MFDBS ’89).

[11] Matúš F. (1991). Abstract functional dependency structures. Theor. Comput. Sci..

[12] Demetrovics J., Libkin L., Muchnik I.B. (1992). Functional Dependencies in Relational Databases: A Lattice Point of View. Discret. Appl. Math..

[13] Levene M. (1995). A Lattice View of Functional Dependencies in Incomplete Relations. Acta Cybern..

[14] Thakor S., Chan T., Grant A. A minimal set of Shannon-type inequalities for functional dependence structures. Proceedings of the 2017 IEEE International Symposium on Information Theory (ISIT).

[15] Chan T., Thakor S., Grant A. A Minimal Set of Shannon-type Inequalities for MRF Structures with Functional Dependencies. Proceedings of the 2018 IEEE International Symposium on Information Theory (ISIT).

[16] Harremoës P. (1993). Time and Conditional Independence; IMFUFA-Tekst, IMFUFA Roskilde University. http://www.harremoes.dk/Peter/afh/afhandling.pdf.

[17] Caspard N., Monjardet B. (2003). The lattices of closure systems, closure operators, and implicational systems on a finite set: A survey. Discret. Appl. Math..

[18] Grätzer G. (2003). General Lattice Theory.

[19] Armstrong W.W. Dependency Structures of Data Base Relationships. Proceedings of the IFIP Congress.

[20] Ullman J.D. (1989). Principles of Database and Knowledge-Base Systems.

[21] Levene M., Loizou G. (1999). A Guide Tour of Relational Databases and Beyond.

[22] Whitman P.M. (1946). Lattices, equivalence relations, and subgroups. Bull. Am. Math. Soc..

[23] Shannon C. (1953). The lattice theory of information. Trans. IRE Prof. Group Inf. Theory.

[24] Dawid A.P. (2001). Separoids: A mathematical framework for conditional independence and irrelevance. Ann. Math. Artif. Intell..

[25] Constantinou P., Dawid A.P. (2017). Extended Conditional Independence and Applications in Causal Inference. Ann. Stat..

[26] Paolini G. (2015). Independence Logic and Abstract Independence Relations. Math. Logic Q..

[27] Pearl J. (1988). Probabilistic Reasoning in Intelligent Systems.

[28] Studený M. (2005). Probabilistic Conditional Independence Structures.

[29] Studený M. (1990). Conditional Independence Relations Have No Finite Complete Characterization. http://citeseerx.ist.psu.edu/viewdoc/download?doi=10.1.1.51.7014&rep=rep1&type=pdf.

[30] Schmidt R. (1994). Subgroup Lattices of Groups.

[31] Matúš F. Infinitely many information inequalities. Proceedings of the 2007 IEEE International Symposium on Information Theory.

[32] Vámos P. (1978). The Missing Axiom of Matroid Theory is Lost Forever. J. Lond. Math. Soc..

[33] Mayhew D., Whittle G., Newman M. (2014). Is the Missing Axiom of Matroid Theory Lost Forever?. Q. J. Math..

[34] Mayhew D., Newman M., Whittle G. (2018). Yes, the “missing axiom” of matroid theory is lost forever. Trans. Am. Math. Soc..

[35] Czédli G. (1982). Factor lattices by tolerance. Acta Sci. Math..

[36] Hermann C. (1973). S-verklebte Summen von Verbänden. Math. Z..

[37] Quackenbush R.W. Planar Lattices. Proceedings of the University of Houston Lattice Theory Conference 1973.

[38] Dilworth R.P. (1950). A decomposition theorem for partially ordered sets. Ann. Math..

[39] Chen C.C., Koh K.M. (1973). A characterization of finite distributive planar lattices. Discret. Math..

[40] Quackenbush G.G.W. (2010). The variety generated by planar modular lattices. Algebra Universalis.

[41] Guille L., Chan T., Grant A. (2011). The Minimal Set of Ingleton Inequalities. IEEE Trans. Inform. Theory.

[42] Paajanen P. (2014). Finite *p*-Groups, Entropy Vectors, and the Ingleton Inequality for Nilpotent Groups. IEEE Trans. Inf. Theory.

[43] Harremoës P. Influence Diagrams as Convex Geometries. http://www.harremoes.dk/Peter/FunctionalDAG.pdf.

